# Global transcriptional profiling between inbred parents and hybrids provides comprehensive insights into ear-length heterosis of maize (*Zea mays*)

**DOI:** 10.1186/s12870-021-02890-1

**Published:** 2021-02-26

**Authors:** Xiangge Zhang, Chenchen Ma, Xiaoqing Wang, Mingbo Wu, Jingkuan Shao, Li Huang, Liang Yuan, Zhiyuan Fu, Weihua Li, Xuehai Zhang, Zhanyong Guo, Jihua Tang

**Affiliations:** 1 National Key Laboratory of Wheat and Maize Crops Science, College of Agronomy, Henan Agricultural University, Zhengzhou, 450018 China; 2 Hubei Collaborative Innovation Center for Grain Industry, Yangtze University, Jingzhou, 433200 China

**Keywords:** Maize, Heterosis, Ear length, Transcriptional regulation, Non-additive expression patterns

## Abstract

**Background:**

Maize (*Zea mays*) ear length, which is an important yield component, exhibits strong heterosis. Understanding the potential molecular mechanisms of ear-length heterosis is critical for efficient yield-related breeding.

**Results:**

Here, a joint netted pattern, including six parent-hybrid triplets, was designed on the basis of two maize lines harboring long (T121 line) and short (T126 line) ears. Global transcriptional profiling of young ears (containing meristem) was performed. Multiple comparative analyses revealed that 874 differentially expressed genes are mainly responsible for the ear-length variation between T121 and T126 lines. Among them, four key genes, *Zm00001d049958*, *Zm00001d027359*, *Zm00001d048502* and *Zm00001d052138*, were identified as being related to meristem development, which corroborated their roles in the superior additive genetic effects on ear length in T121 line. Non-additive expression patterns were used to identify candidate genes related to ear-length heterosis. A non-additively expressed gene (*Zm00001d050649*) was associated with the timing of meristematic phase transition and was determined to be the homolog of tomato *SELF PRUNING*, which assists *SINGLE FLOWER TRUSS* in driving yield-related heterosis, indicating that *Zm00001d050649* is a potential contributor to drive heterotic effect on ear length.

**Conclusion:**

Our results suggest that inbred parents provide genetic and heterotic effects on the ear lengths of their corresponding F_1_ hybrids through two independent pathways. These findings provide comprehensive insights into the transcriptional regulation of ear length and improve the understanding of ear-length heterosis in maize.

**Supplementary Information:**

The online version contains supplementary material available at 10.1186/s12870-021-02890-1.

## Background

Heterosis is a phenomenon in which F_1_ hybrid progenies exhibit superior performances compared with those of their parents [1–3]. It is used for hybrid crop breeding, which has greatly increased the productivity of many crops worldwide [4, 5]. The successful exploitation of heterosis has also led researchers to determine its essential features. From the formation of two hypotheses (dominance and over-dominance) [6, 7] to the identification of genetic components [8–10], as well as comprehensive analyses of genomes, transcriptomes and metabolomes [11–16], tremendous efforts have been made to elucidate the mechanisms responsible for heterosis. However, the underlying molecular mechanisms remain poorly understood.

Transcriptional regulation plays roles in various aspects of plant growth and development. Variation in transcriptional regulation promotes phenotypic diversity in all species [17] and, thus, is a potential source of heterosis that could explain the differences between F_1_ hybrids and their parental lines. Many transcriptome analyses between hybrids and inbred lines have been carried out in both maize (*Zea mays*) and rice (*Oryza sativa*), and a great number of differentially expressed genes (DEGs) were found in F_1_ hybrids compared with their parents [4, 18–20]. Thus, heterosis was assumed to result from the global variation in gene expression between hybrids and inbred lines. However, several kinds of gene expression modes were observed in F_1_ hybrids: mid-parent (MP) (additivity), high and low parent (high and low parent dominance, respectively), above the high parent (over-dominance) and below the low parent (under-dominance) [21]. These data revealed that in hybrids, some genes exhibit non-additive expression patterns (not the expected MP level), which suggested a potential association with heterosis [22–24]. These expression differences may be caused by allele-specific expression (ASE), which refers to the characteristic of preferentially expressing one parental allele in the hybrid owing to variations in regulatory sequences from the parental genome [25, 26]. Trans- and cis-regulation frequently exist in different parental lines, and they might be responsible for inducing ASE in hybrids [17, 27]. Consequently, analyzing transcriptional regulation is a valuable strategy for untangling the molecular basis of heterosis.

The measurement of heterosis involves a specific trait. Moreover, heterotic level is highly variable depending on the species, the cross parents and the trait(s) of interest [18]. Maize is an important food crop worldwide, and it exhibits superior heterosis for a wide range of traits. In addition, its inbred lines have been classified into several “heterotic groups” on the basis of their heterotic level [28, 29]. Generally, crosses of parents within heterotic groups produce less heterosis than crosses of parents in different groups. This suggested that the inbred lines in each group may have specific exclusive properties that contribute to heterosis. Thus, between-group crosses are more likely to produce greater heterosis. Maize ear length is a representative trait with a superior heterotic level, and it contributes greatly to grain yield [30]. A thorough knowledge of the transcriptional regulation of ear-length heterosis will aid in understanding the molecular basis of heterosis.

Maize ear length is predetermined, to some extent, by the activity of the ear primordium. As the ear primordium (meristem) differentiates, the visible young ear gradually elongates, revealing heterosis, and it is positively correlated with the final ear length [30]. In this study, two specific maize lines, T121 and T126, with long and short ears, respectively, were identified. When crossed with other lines, T121 line, compared with T126 line, produced a series of hybrids with longer ears. However, equal ear-length heterosis was observed in their corresponding hybrids. With these two specific maize lines, we performed comprehensive transcriptional profiling of young ears using a joint netted pattern to determine the underlying cause of long ear in T121 line and to gain insights into ear-length heterosis.

## Results

### The performance of ear length in different inbred-hybrid triplets

**Fig. 1:**
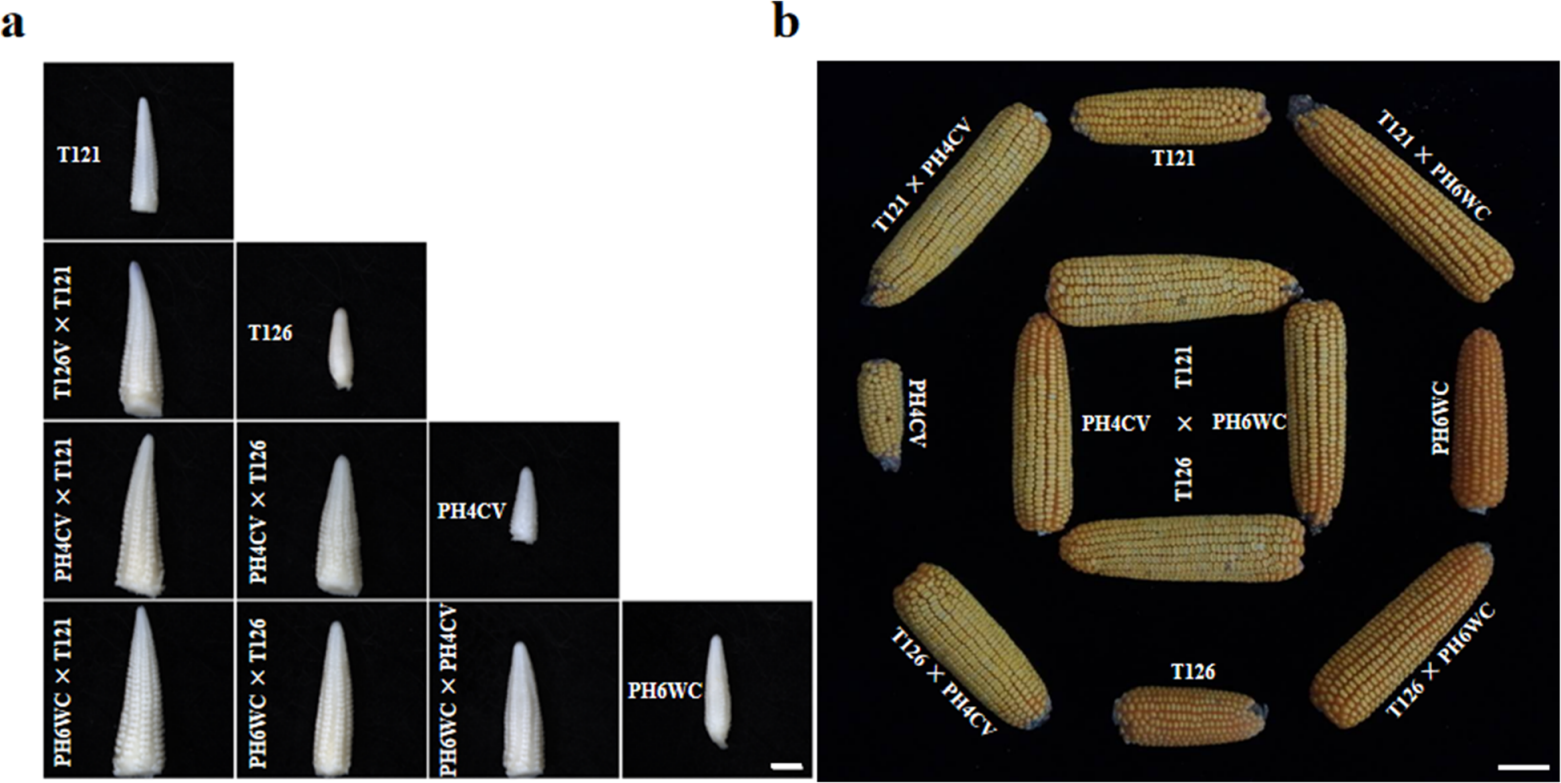
Phenotypic observations of maize ears from six parent-hybrid triplets. **a** Morphological comparisons of the young ears at the 13-leaf stage. Bar = 1 mm. **b** Phenotypic variations in the ears at the mature stage. Bar = 6 cm

Ear-length heterosis in maize is a very striking phenomenon resulting from a cross of two distinct inbred lines. To explore ear-length heterosis, we selected two specific inbred lines (T121 and T126) with long and short ears, respectively. Additionally, two other inbred lines (PH4CV and PH6WC) were used to form a joint netted pattern (Fig. S1) that included six parent-hybrid triplets to adequately analyze ear-length heterosis. During maize ear differentiation, the young ear gradually elongates and becomes visible. Moreover, the elongation capability of the growth cone determines the final ear length to some extent. Here, we compared the morphologies of young ears of hybrids and their inbred parents at the 13-leaf stage when young ears were initially apparent. The young ears of T121 line were longer than those of T126 line. Moreover, the F_1_ hybrids generated by T121 crosses (T121 × PH4CV and T121 × PH6WC) had longer young ears than those generated by corresponding T126 crosses (T126 × PH4CV and T126 × PH6WC) (Table 1, Fig. 1a). In addition, the lengths of young ears from each inbred parent were less than those of their F_1_ hybrids (Table 1, Fig. 1a), indicating that ear-length heterosis had already emerged.

**Table 1:** Ear-length performance in different maize inbred-hybrid triplets

Lines	Yong ear		Mature ear	
	Ear length (mm)	MPH	Ear length (cm)	MPH
T121	3.79 ± 0.08^e^		19.52 ± 1.18^e^	
T126	2.58 ± 0.06^g^		13.52 ± 0.83^g^	
PH4CV	2.42 ± 0.08^g^		11.26 ± 1.08^h^	
PH6WC	3.26 ± 0.05^f^		17.03 ± 1.03^f^	
T121 × PH4CV	5.37 ± 0.15^b^	72.95%	25.53 ± 1.48^b^	65.89%
T126 × PH4CV	4.53 ± 0.06^d^	81.20%	20.06 ± 1.35^e^	61.90%
T121 × PH6WC	5.89 ± 0.17^a^	67.09%	27.01 ± 1.56^a^	47.80%
T126 × PH6WC	5.11 ± 0.11^bc^	75.00%	22.51 ± 1.02^d^	47.36%
T121 × T126	4.84 ± 0.07^c^	51.96%	23.82 ± 1.28^c^	44.19%
PH4CV × PH6WC	4.68 ± 0.09^cd^	64.79%	22.78 ± 1.19^d^	61.05%

Values in the ear length columns are means ± standard deviations; the superscripted letters represent the significance by least significance difference (LSD) at 0.05 level. MPH, MP heterosis

At the maturation stage, we measured the final ear lengths of all lines (inbred and hybrid). T121 line, a long-ear inbred line, had an ear length that reached 19.52 ± 1.18 cm and was much longer than that of the T126 (13.52 ± 0.83 cm) line (Table 1, Fig. 1b). The six F_1_ hybrids, T121 × PH4CV, T121 × PH6WC, T126 × PH4CV, T126 × PH6WC, T121 × T126 and PH6WC × PH4CV, exhibited MP heterosis for ear length (Table 1). Interestingly, the hybrids produced by T121 line (long ear), T121 × PH4CV and T121 × PH6WC, had longer ears than those produced by the short ear line T126 (Fig. 1b). However, for the MP heterosis, there was no significant difference between the other corresponding hybrids, such as T121 × PH4CV vs T126 × PH4CV **(**Table 1**)**. These data indicate that T121 line makes a superior contribution to ear length than T126 line, but this is not a result of ear-length heterosis.

### Transcriptome profiles of maize young ears among four inbred parents and six F_1_ hybrids

To understand the comprehensive transcriptional regulation of maize ear-length heterosis, young ears of four inbred parents and six F_1_ hybrids were used to perform RNA-sequencing (RNA-seq) analysis at the 13-leaf stage. In total, 332,543,189 raw reads were generated, ranging from 13.87 million to 21.76 million per library (Table 2). After filtering, 320,828,384 clean reads, accounting for 96.48% of the total, were maintained (Table 2). Based on the B73 maize reference genome (Version 4), the average unique mapping rate was 85.47%, with a range from 79.03 to 87.93% (Table 2). Moreover, two biological replicates were in close agreement (Fig. S2). Finally, 25,199 unique genes were identified in all lines (Table S1). The RNA-seq data is available for further analyses of transcriptional regulation.

**Table 2:** RNA-seq read information for all the maize samples

Samples	Raw reads	Clean reads	Retention rate	Mapped reads	Mapped rate
T121–1	13,871,140	13,443,019	96.91%	11,622,834	86.46%
T121–2	16,291,424	15,500,533	95.15%	13,091,750	84.46%
T126–1	17,965,773	17,397,315	96.84%	14,993,006	86.18%
T126–2	18,368,761	17,927,296	97.60%	15,485,598	86.38%
PH4CV-1	14,658,208	13,170,436	89.85%	10,408,596	79.03%
PH4CV-2	21,755,428	19,974,317	91.81%	16,185,189	81.03%
PH6WC-1	15,884,601	15,718,724	98.96%	13,711,443	87.23%
PH6WC-2	14,723,316	14,399,270	97.80%	12,569,123	87.29%
T121 × PH4CV-1	14,077,347	13,499,094	95.89%	11,445,882	84.79%
T121 × PH4CV-2	16,779,865	16,290,901	97.09%	14,021,578	86.07%
T121 × PH6WC-1	18,542,048	18,152,498	97.90%	15,750,923	86.77%
T121 × PH6WC-2	19,966,028	19,316,617	96.75%	16,480,938	85.32%
T121 × T126–1	15,417,581	15,241,180	98.86%	13,401,570	87.93%
T121 × T126–2	16,935,225	16,415,063	96.93%	14,049,652	85.59%
T126 × PH4CV-1	15,196,264	14,513,192	95.51%	12,233,170	84.29%
T126 × PH4CV-2	19,608,143	18,982,604	96.81%	16,230,126	85.50%
PH4CV × PH6WC-1	14,888,697	14,495,750	97.36%	12,502,584	86.25%
PH4CV × PH6WC-2	15,276,520	14,917,001	97.65%	12,855,471	86.18%
T126 × PH6WC-1	17,312,865	16,694,768	96.43%	14,243,976	85.32%
T126 × PH6WC-2	15,023,955	14,778,806	98.37%	12,903,376	87.31%
Total	332,543,189	320,828,384	96.48%	274,186,785	85.47%

### Global transcriptome changes from inbred parents to their hybrids

**Fig. 2:**
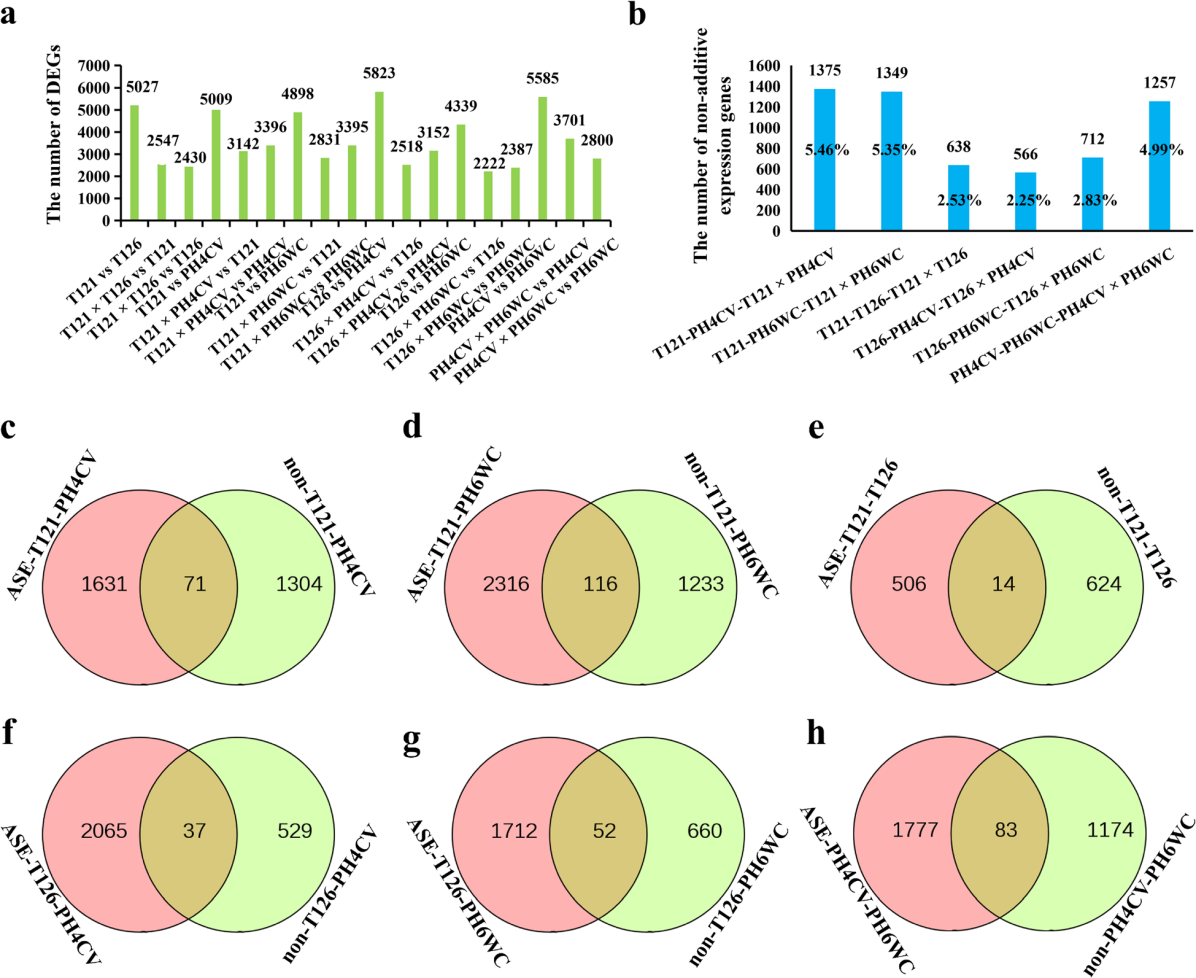
Global transcriptome changes from maize inbred parents to their hybrids. **a** The numbers of DEGs between lines in the six triplets. **b** The numbers of non-additively expressed genes in each triplet. **c**–**h** Venn diagram comparisons between genes having ASE and non-additively expressed genes for triplets T121-PH4CV, T121-PH6WC, T121-T126, T126-PH4CV, T126-PH6WC and PH4CV-PH6WC, respectively. ASE-, allele-specific expression; non-, non-additive expression

Variation in gene expression is closely associated with phenotypic diversity. Thus, a series of transcriptional changes should occur from two inbred parents to one hybrid. For the T121–T126–T121 × T126 triplet, 64.97% of the genes in T121 × T126 hybrid kept their expression levels within the parental range, whereas the expression levels of the remaining genes (35.03%) were out of this rang (Table 3). This data indicated that the hybrids had the sufficient potential to surpass the two parents. Using a differential expression analysis, 5027 DEGs were identified between T121 and T126, 2547 DEGs were identified between T121 × T126 and T121, and 2431 DEGs were identified between T121 × T126 and T126 (Fig. 2a; Table S2). Thus, the number of DEGs between a hybrid and one parent (T121 or T126) was less than that between the two parents. Moreover, similar scenarios, including the ranges of the gene expression levels and the numbers of DEGs, were found in other parent-hybrid triplets (Tables 3, S2; Fig. 2a). Thus, some transcriptional regulatory mechanisms appeared to be universal and common in the production of hybrids from inbred parents.

**Table 3:** Variations in expression levels from the two parents to the F_1_ hybrid in each maize triplet

Triplets	Within parental rang	Out of parental rang
T121-T126-T121 × T126	16,371 (64.97%)	8828 (35.03%)
T121-PH4CV-T121 × PH4CV	14,509 (57.58%)	10,690 (42.42%)
T121-PH6WC-T121 × PH6WC	14,980 (59.45%)	10,219 (40.55%)
T126-PH4CV-T126 × PH4CV	17,027 (67.57%)	8172 (32.43%)
T126-PH6WC-T126 × PH6WC	14,874 (59.03%)	10,325 (40.97%)
PH4CV-PH6WC-PH4CV × PH6WC	15,420 (61.19%)	9779 (38.81%)

The figure outside the brackets represents the number of genes and those in the brackets represents its corresponding proportion

In F_1_ hybrids, there are two gene expression patterns: additive and non-additive. We performed a non-additive expression analysis for the six parent-hybrid triplets. In total, 1375, 1349, 638, 566, 712 and 1257 non-additive genes were identified for T121–PH4CV–T121 × PH4CV, T121–PH6WC–T121 × PH6WC, T121–T126–T121 × T126, T126–PH4CV–T126 × PH4CV, T126–PH6WC–T126 × PH6WC and PH4CV–PH6WC–PH4CV × PH6WC, respectively (Fig. 2b; Table S3). A small proportion (< 6%) of non-additive patterns appeared in all F_1_ hybrids (Fig. 2b), indicating that most genes displayed an additive pattern in F_1_ hybrids. The additive pattern represents the expected MP level, whereas the non-additive pattern significantly deviates from the MP level. Thus, the non-additive genes in each triplet may have contributed to ear-length heterosis.

In hybrids, ASE frequently exists, increasing the plasticity of gene expression governed by diverse alleles from two parents, and this may be the reason that non-additive patterns appear in F_1_ hybrids. Thus, we analyzed genes having ASE in all the parent-hybrid triplets and then compared them with non-additively expressed genes identified in the same triplet. Quite a number of genes harboring ASE were detected in hybrids (Table S4). However, few of them were non-additively expressed (Fig. 2c–h). For instance, in the T121 × PH4CV hybrid, 1702 genes having ASE were identified, but only 71 exhibited non-additive expression patterns (Fig. 2c). These results indicated that in F_1_ hybrids, ASE might have a limited contribution to the production of non-additive expression-related variation.

### The major genes responsible for the ear-length variation between T121 and T126 lines

**Fig. 3:**
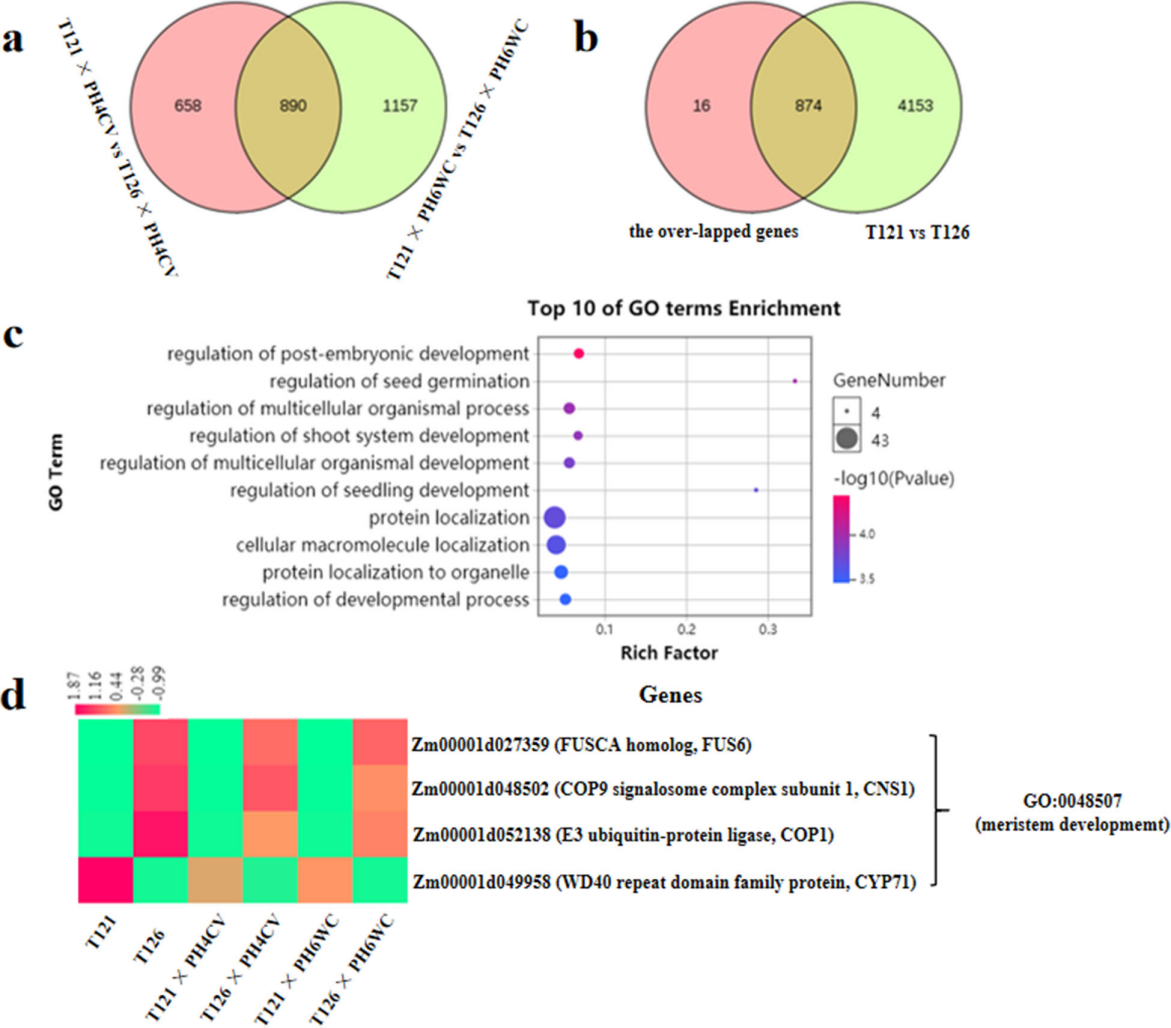
Identification of the major genes responsible for the ear-length variation between T121 and T126 lines. **a** Venn diagram comparison of two groups of DEGs between the corresponding F_1_ hybrids derived from the T121 and T126 lines. **b** Venn diagram comparison of the overlapped genes in A and the DEGs between the T121 and T126 lines. **c** The top 10 GO term analysis of the shared genes in b. **d** The candidate DEGs in meristem-related GO terms

T121 line produces longer ears than T126 line at the 13-leaf stage. A transcriptional level analysis of young ears revealed a large number of DEGs (5027) between T121 and T126 lines. However, it was difficult to determine the major genes responsible for the ear-length variation. Nevertheless, compared with T126 line, T121 had a longer ear and might pass this advantage to its F_1_ hybrid. When T121 and T126 were hybridized with the other parents (PH4CV and PH6WC), the former produced F_1_ hybrids with longer ears compared with the latter. Consequently, we performed a differential expression analysis between the corresponding F_1_ hybrids, T121 × PH4CV vs T126 × PH4CV and T121 × PH6WC vs T126 × PH6WC (Table S2). In total, 890 DEGs were found to overlap between the two groups (Fig. 3a). We compared these overlapped genes with the DEGs identified between lines T121 and T126. As expected, they shared many common genes (874) (Fig. 3b), which suggested that these genes take part in the regulation of ear elongation and are mainly responsible for the ear-length variation between T121 and T126 lines.

Gene ontology (GO) enrichment analysis was performed to identify some major terms related to ear length, as well as the key genes implicated in ear-length heterosis. A total of 1672 GO terms were enriched for these genes in biological process (Table S5). Furthermore, the top 10 GO terms were investigated, and they revealed several terms related to development, such as GO:0048582 (regulation of post-embryonic development) and GO:0048831 (regulation of shoot system development) (Fig. 3c; Table S5). Among these terms, four genes (Fig. 3d), *Zm00001d027359* (FUSCA homolog, *FUS6*), *Zm00001d048502* (COP9 signalosome complex subunit 1, *CNS1*), *Zm00001d052138* (E3 ubiquitin-protein ligase, *COP1*) and *Zm00001d049958* (WD40 repeat domain family protein, *CYP71*), were found to also belong to GO:0048507 (meristem development), and they may make major contributions to ear-length variation.

### Non-additively expressed genes contributing to ear-length heterosis

**Fig. 4:**
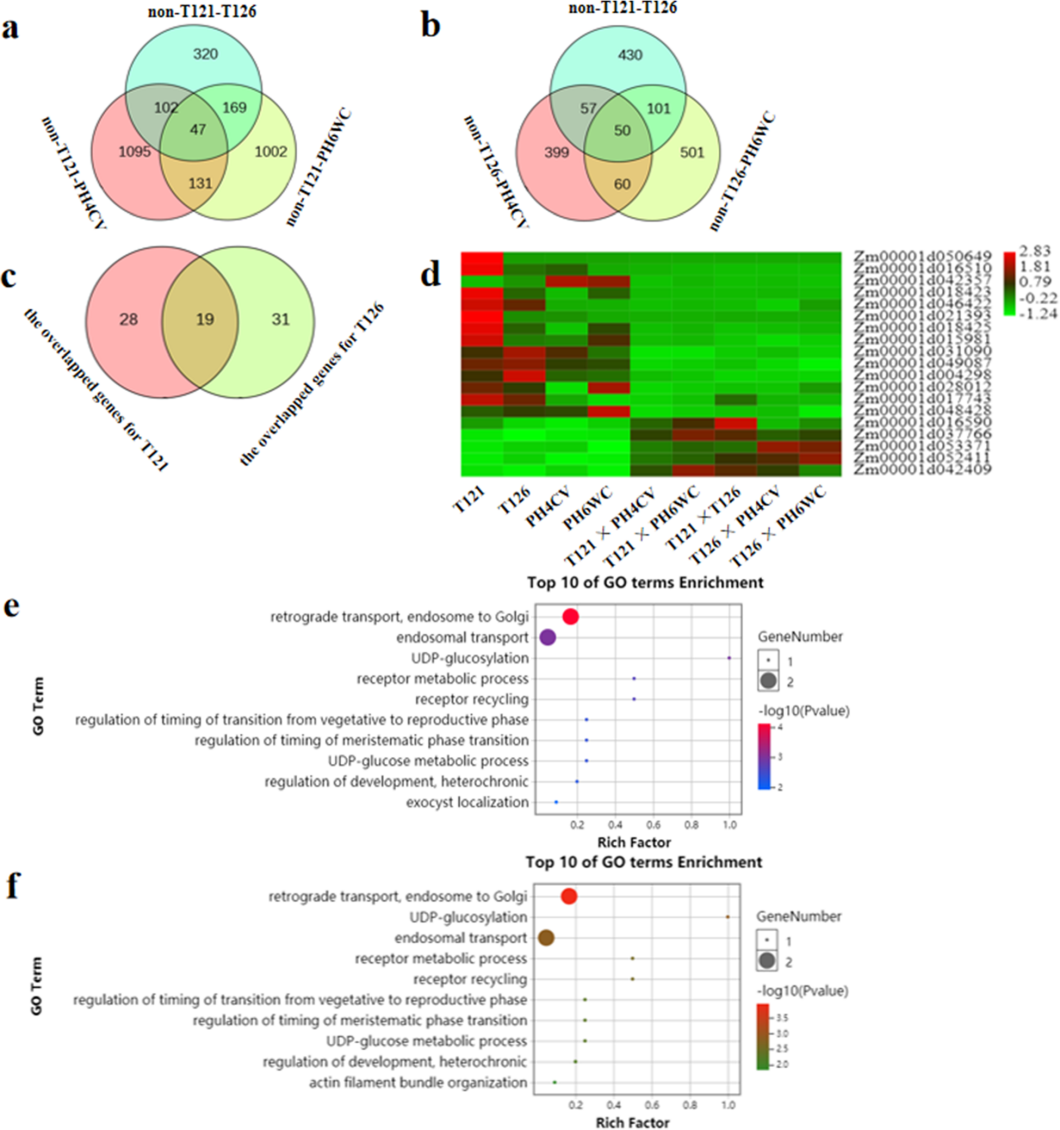
Identification of non-additively expressed genes potentially contributing to maize ear-length heterosis. **a**, **b** Venn diagram comparisons among the non-additively expressed genes of the triplets including the T121 and T126 lines, respectively. Non-, non-additive expression. **c** Venn diagram comparison between the overlapped genes for T121 line in a and T126 line in b. **d** The expression profiles of the common genes in c. **e**, **f** The top 10 GO term analysis of the candidate genes related to ear-length heterosis of the T121 and T126 lines, respectively

Non-additively expressed genes may be potential sources of heterosis [31]. To identify promising potential genes that contribute to ear-length heterosis derived from T121 (or T126) line, we made multiple comparisons of non-additively expressed genes in these parent-hybrid triplets. For T121 line, 47 non-additively expressed genes overlapped among hybrids produced by T121 × PH4CV, T121 × PH6WC and T121 × T126 (Fig. 4a). Whereas, for 126 line, 50 common non-additively expressed genes were identified among hybrids produced by T126 × PH4CV, T126 × PH6WC and T121 × T126 (Fig. 4b). These genes should be involved in ear-length heterosis, because the ear lengths of these F_1_ hybrids all surpassed the MP values. Moreover, 19 genes were shared (Fig. 4c, d), and these genes displayed non-additive expression patterns in all hybrids, suggesting that they had a potential to contribute to ear-length heterosis. GO enrichment analysis revealed that the top 10 GO terms for T121 and T126 lines were highly similar (Fig. 4e, f; Tables S6, 7), suggesting that there are some common components of the mechanism underlying ear-length heterosis. Among the common GO terms, GO:0048506 (regulation of timing of meristematic phase transition) and GO:0048510 (regulation of timing of transition from vegetative to reproductive phase) were associated with meristem, and a shared gene, *Zm00001d050649* (*ZCN2*), may be responsible for the ear-length heterosis.

### Validation of candidate gene expression by quantitative real-time PCR

**Fig. 5:**
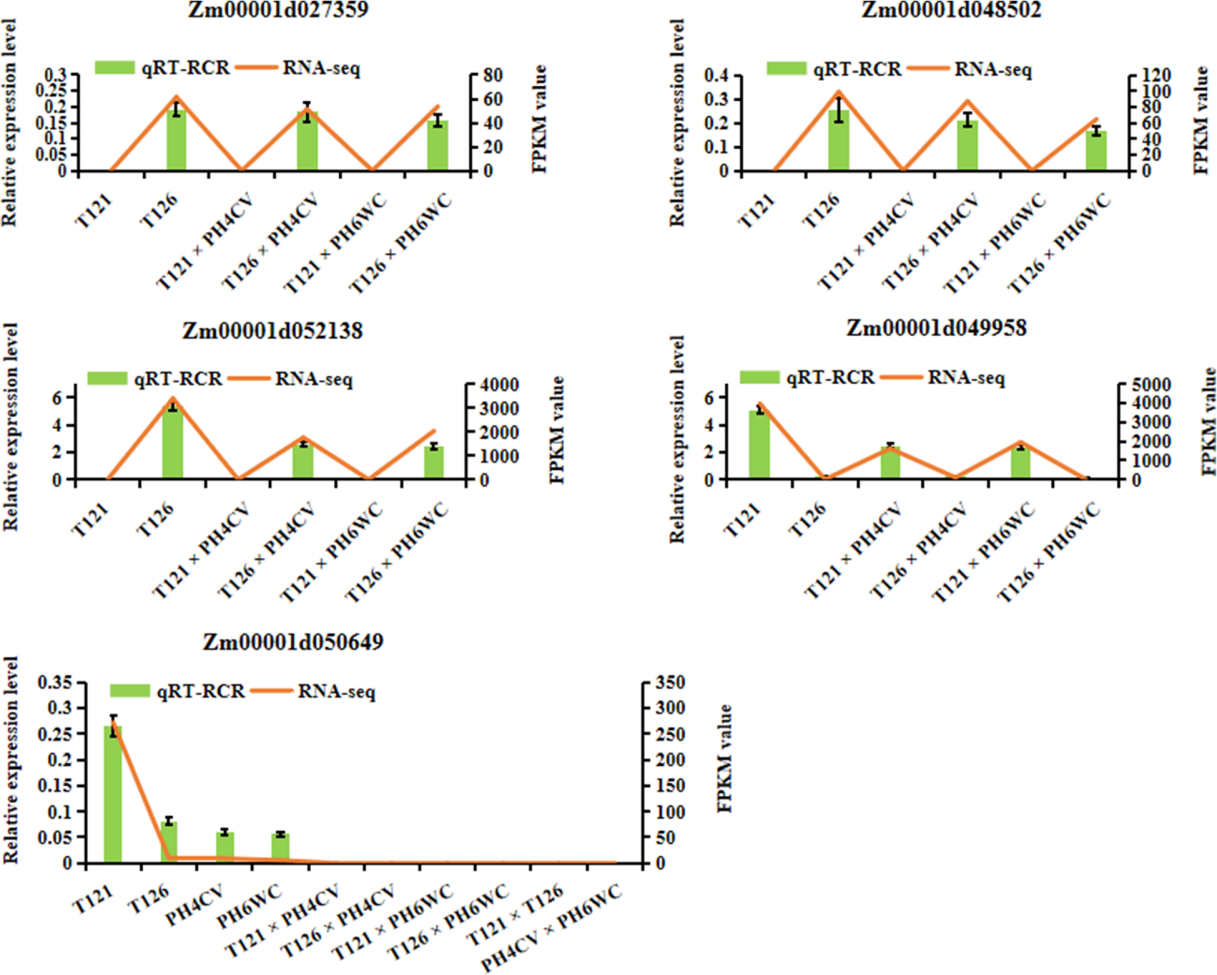
The relative expression levels of five candidate maize genes as assessed by qRT-PCR. The first four graphs show the four DEGs involved in additive genetic effects on ear length; the last graph shows a non-additively expressed gene involved in heterotic effects on ear length

The application of RNA-seq technology has greatly enhanced the global understanding of transcriptional regulatory networks. To verify the accuracy of RNA-seq analysis, we performed a quantitative real-time PCR (qRT-PCR) analysis of five candidate genes, including four DEGs having additive genetic effects on ear length, *Zm00001d027359*, *Zm00001d048502*, *Zm00001d052138* and *Zm00001d049958*, and one non-additively expressed gene having heterotic effects on ear length, *Zm00001d050649*. Primers were designed to specifically amplify each of the five genes (Table S8). These primers were used to conduct qRT-PCR on three biological replications of RNA from re-prepared samples. All the assayed genes showed expression patterns similar to those determined by RNA-seq (Fig. 5), verifying the reliability of our RNA-seq analysis.

## Discussion

### Two independent pathways of genetic and heterotic effects on ear length in maize

Three major hypotheses, dominance [6, 32], over-dominance [7, 33] and epistasis [34, 35], have served as the foundation for exploring the genetic and molecular causes of heterosis. The core notions are complementation within alleles, interactions within alleles and interactions between alleles, respectively [2]. All of them highlight the potential contributions of alleles (preferably considered as genetic loci) from two parental inbred lines to their F_1_ hybrid. Indeed, conventional genetic loci refer to quantitative trait loci (QTL) for per se traits that control the additive genetic effect for trait performance, while heterotic effects are determined by special genetic loci, defined as QTL for trait heterosis, based on MP heterosis [36]. Using recombinant inbred lines and immortalized F_2_ populations, some researchers have identified large numbers of QTL for per se traits and QTL for trait heterosis, respectively. Interestingly, very few of the two kinds overlaped, indicating that two independent pathways are responsible for their respective contributions to trait performance [10, 37]. However, quite a few overlapped loci were revealed in another study [9]. More information is needed to determine the relationships between genomics of a per se trait and those involved in heterosis of the same trait.

Gene expression is a complex process involving a series of transcriptional regulations that affect an individual’s phenotype [17]. Transcriptional regulation plays a role in explaining the molecular mechanisms of heterosis that benefit F_1_ hybrid individuals with a superior trait performance compared with the parental inbred individuals [19, 38]. Transcriptome profiles of two inbred lines and their hybrids have been determined to investigate variations in global gene expression. Consistent with many previous studies [39–42], there were more DEGs between the two parents than between each parent and the hybrid in all triplets, and only a few genes (< 6%) displayed non-additive patterns (Fig. 2b). Thus, the prevailing additive pattern appears to limit the difference between one parent and the hybrids and makes a limited contribution to heterosis in hybrids. Additionally, T121 line produced longer ears than T126 line, which indicated that T121 line had a superior additive genetic effect on ear length. However, their corresponding hybrids, such as T121 × PH4CV vs T126 × PH4CV, produced nearly equal levels of heterosis for ear length (Table 1). Thus, the additive genetic effect (parental variation) and heterotic effect appeared to be uncorrelated. Similarly, the genetic distance between two parents is a limited predictor of heterosis in their F_1_ hybrids [18].

Indeed, the ear lengths of T121 × T126 hybrids easily surpassed that of T121 line (extremely long ear). This was attributed to ear-length heterosis. Thus, the DEGs between T121 × T126 and T121 should be involved in ear-length heterosis, which increased the ear length compared with that of T121 line. However, some of these genes were also identified as being responsible for the ear-length variation between T121 and T126 lines, but their variation trends were opposite (up−/down-regulation) (Fig. S3). This indicated that these shared genes may not play roles in the ear-length heterosis. In this context, two inbred parents would provide the genetic and heterotic effects on the ear lengths of their F_1_ hybrids through two independent pathways. The superior performance of the hybrid over that of the better inbred parent benefits from the altered regulation of specific genes having non-additive expression patterns.

### A possible pathway resulting in the non-additive expression pattern from inbred parents to their hybrids

From two inbred parents to one hybrid, it is tempting to infer that some new transcriptional regulations (non-additive expression patterns) contribute to heterosis. Determining the pathway responsible for the non-additive expression pattern may help to elucidate the mechanisms of heterosis.

Most transcriptional variation may be caused by sequence variation in regulatory regions of genes (cis-regulation) or by functional variations in a regulator (trans-regulation) [43]. In hybrids, all genes consist of a pair of alleles derived from two parental inbred lines, respectively. Usually, the biased expression of alleles (ASE) takes place in some genes. If cis-regulation is present, then the allelic expression is expected to be additive in the hybrids, resulting in an additive expression pattern. In this study, we found that few genes having ASE present the non-additive expression pattern in all parent-hybrid triplets (Fig. 2c–h). Thus, this indicated that ASE is mainly caused by cis-regulation and makes a limited contribution to the non-additive expression pattern. Indeed, numerous studies [44–47] have revealed that cis-regulation plays major roles in the regulation of allelic expression in the hybrids of many species, and this may result in the production of large numbers of additively expressed genes.

Trans-regulation also occurs widely among regulatory networks, and it regulates the expression of many genes [17]. A deficiency in trans-acting factors in one parent leads to the differential expression of their target genes between parents. However, because F_1_ hybrids have the same genetic background, the sharing of trans-acting factors facilitates the balanced expression of allelic genes, resulting in non-additive expression patterns. Here, we compared the two blocks: non-additively expressed genes in F_1_ hybrids and DEGs between two parents. Several common genes were discovered in each triplet (Fig. S4). For example, in the T121–PH4CV–T121 × PH4CV triplet, 395 genes overlapped between the two blocks (Fig. S4a). This revealed that trans-regulation may cause the variation in gene expression between two parents and produce the non-additive expression pattern in F_1_ hybrids. However, such genes would be responsible for the variation in ear length between the two parents (additive genetic effect) but would not contribute to ear-length heterosis.

Indeed, numerous isolated non-additively expressed genes were not differentially expressed between two inbred parents and presented non-ASE patterns in corresponding F_1_ hybrids (Figs. 2b, S4). One possible scenario is that cis- and trans-interactions occur. If the cis-regulated alleles also harbor variations in functions (trans-regulation), then non-additive expression patterns would be produced. An excellent example has been reported in tomato hybrids, in which yield was improved by fine-tuning the expression of a transcription factor (MADS-box) and its trans-effects on the target alleles [48]. Alternatively, two or more trans-acting factors may combine to activate or suppress the expression of the target alleles in the F_1_ hybrids, leading to a non-additive expression pattern. For example, two maize transcription factors, B and Pl, interact to up-regulate the expression of genes *A1*, *A2* and *Bz1*, which control anthocyanin production [49]. An inbred line with a nonfunctional *b* or *pl* allele displays a green phenotype owing to the low, or absent, expression of genes *A1*, *A2* and *Bz1*, but a hybrid with *B*/*b Pl*/*pl* alleles has high expression levels for genes *A1*, *A2* and *Bz1* and a red phenotype [46]. Thus, once two parents are crossed, some specific interactions, rather than either cis- or trans-regulation, play major roles in generating the non-additive expression patterns found in hybrids, contributing to heterosis.

### Key genes having superior additive genetic effects on ear length in T121 line

One inbred line often transfers its excellent characteristic to its progeny, including F_1_ hybrids, and this can be attributed to its superior additive genetic effect. In this study, T121 line produced an extremely long ear, and its ear length was far greater than that of T126 line. Likewise, its F_1_ hybrids exhibited longer ears compared with those of T126 line, indicating that T121 line had a superior additive genetic effect on ear length.

Maize ear development arises from axillary meristem, which can greatly influence ear size [50]. Here, we identified four candidate genes related to meristem development, *Zm00001d027359* (*FUS6*), *Zm00001d048502* (*CNS1*), *Zm00001d052138* (*COP1*) and *Zm00001d049958* (*CYP71*). Interestingly, FUS6/CNS1 (FUS6 also called CNS1) and COP1 were originally found to act together in the photomorphogenesis of shoot apical meristem in Arabidopsis [51–53]. The COP9 signalosome (CSN) is a multifunctional protein complex composed of eight subunits (CSN1–8) in higher eukaryotes, such as plants, which regulates the activity levels of the cullin-RING ligase families of E3 ubiquitin ligase complexes [51]. COP1 acts as an E3 ubiquitin ligase and translocates into the nucleus in a CSN-dependent manner, where it suppresses photomorphogenesis by promoting the degradation of positive of photomorphogenic regulators in the darkness [54, 55]. Upon light exposure, the nuclear COP1 is rapidly depleted, thus alleviating its suppression of photomorphogenic development [54]. CSN and COP1 are involved in a range of plant growth and developmental processes [53, 55–57]. Therefore, the lower expression levels of *Zm00001d027359* (*FUS6*/*CNS1*), *Zm00001d048502* (*CNS1*) and *Zm00001d052138* (*COP1*) in T121, T121 × PH4CV and T121 × PH6WC individuals (Figs. 3d, 5) accelerate ear growth and the rapid growth of plants after releasing photomorphogenetic suppression.

Additionally, Arabidopsis CYP71 is a unique immunophilin with a WD40 domain, and it interacts with histone H3 to regulate gene expression patterns that determine plant organogenesis [58, 59]. The *CYP71* gene is preferentially expressed in meristem and other actively dividing tissues, and a loss of CYP71 function causes the arrest of apical meristem development [58, 59]. Similarly, higher expression levels of *Zm00001d049958* (*CYP71*) in T121 line and its progeny (Figs. 3d and 5) was conducive to ear growth. In this context, a possible scenario is that the expression of *Zm00001d049958* (*CYP71*) in maize axillary meristem suppresses the expression of *Zm00001d027359* (*FUS6*/*CNS1*), *Zm00001d048502* (*CNS1*) and *Zm00001d052138* (*COP1*) genes and then promotes ear elongation. Overall, these four genes may be responsible for the additive genetic effects on ear length in T121 line.

### The potential contributors to heterosis for the ear length

The performance of F_1_ hybrids mainly benefits from two aspects: the genetic and heterotic effects derived from two parents. Heterosis is specific to different traits and may be attributed to specific loci for a particular trait [46]. Independent of the loci, drastic transcriptional variations in key genes must take place in hybrids. Genes harboring non-additive expression patterns have been studied owing to their huge contributing potential to heterosis [31, 60, 61].

Maize ear length is an important agronomic trait that often exhibits super heterosis [46]. In this study, we analyzed global transcriptomes of young maize ears from six parent-hybrid triplets derived from four inbred lines. The *Zm00001d050649* (*ZCN2*) gene displayed a non-additive expression pattern in all the triplets and belonged to GO:0048506 (regulation of timing of meristematic phase transition), implying its contribution to ear-length heterosis. Maize *ZCN2* is a member of the *TERMINAL FLOWER1* (*TFL1*)-like gene family, which is highly conserved in plants and is thought to function in the maintenance of meristem indeterminacy [62]. In Arabidopsis, TFL1 and FLOWERING LOCUS T (FT) are two antagonistic integrators of the floral transition pathways that function in repressing and promoting flowering, respectively [63–65]. The tomato *SINGLE FLOWER TRUSS* gene, an ortholog of Arabidopsis *FT*, drives the heterosis for yield in an over-dominant pattern [66]. These heterotic effects depend on the genetic background having a mutation in *SELF PRUNING* (*SP*), an ortholog of Arabidopsis *TFL1*. If plants carry a functional *SP* gene, then heterosis is eliminated [66, 67]. This suggested that the *sp* gene is a required contributor that drives heterosis for yield in tomato. Like the tomato *sp* gene, lack of expression of the homologous maize *ZCN2* gene in hybrids (Fig. 5) contributes to ear-length heterosis.

## Conclusions

In this study, multiple comparative analyses of the transcriptional profiles of six parent-hybrid triplets revealed that the genetic and heterotic effects on ear length in maize contribute to the performance of F_1_ hybrids through two independent pathways. Four key genes, *Zm00001d049958* (*CYP71*), *Zm00001d027359* (*FUS6*/*CNS1*), *Zm00001d048502* (*CNS1*) and *Zm00001d052138* (*COP1*), were identified as being responsible for the superior additive genetic effects on ear length in T121 line. Cis- and trans-regulatory interactions mainly caused the emergence of non-additive expression patterns in F_1_ hybrids, providing the potential to drive ear-length heterosis. The lack of expression of a non-additively expressed gene, *Zm00001d050649* (*ZCN2*) was identified as potentially contributing to ear-length heterosis just as its homologous tomato *SP* gene contributes to yield heterosis. This will lead to investigations of the mechanism behind the silencing of *Zm00001d050649* (*ZCN2*) in F_1_ hybrids, which will help further elucidate the mechanisms of heterosis. The present work provides insights into the transcriptional regulation of the maize ear-length characteristic from two parents to one hybrid. These findings improve our understanding of ear-length heterosis in maize.

## Materials and methods

### Experimental design and plant growth

Four maize inbred lines were selected in this study. T121 and T126 are inbred lines having long and short ears, respectively, which were derived from our breeding lines. The other two inbred lines, PH4CV and PH6WC, are the two parents of the excellent hybrid ‘Xianyu 335’. Then, inbreds were crosses to each other following a half-diallel (without reciprocals) design that resulted in a joint netted pattern comprising six F_1_ hybrids (Fig. S1).

The four parental inbred lines and six F_1_ hybrids were planted in a specially designed plot. The plot consisted of 4-m-long rows separated by 1-m spaces between each row, with 15 individuals planted per row. The five-row interval planting was performed for each line with two replicates. In addition, inbred and hybrid lines were separated. The 5th and 10th leaves of uniform individuals were labelled in the field. These experiments were carried out at the Scientific & Educational Park of Henan Agricultural University, Yuanyang, China.

### Phenotypic heterosis for ear length

In maize, an axillary meristem forms at each stalk node beginning at the base of the stalk and continuing toward the top except for the upper six to eight nodes of the plant. The maize axillary meristem initiates ear development and only the upper one or two ear shoots ultimately become the harvestable (final) ears. The uppermost (final) ear is normally located at the 12th to 14th stalk node, corresponding to the 12th to 14th leaf [68]. In practice, at the 13-leaf stage, young ears approximately 2–5 mm in length were initially visible and easily segregated. Young ears of 10 individuals per line were fixed in FAA composed of 5% formaldehyde (40% v/v), 5% acetate and 90% alcohol (75% v/v). Their morphologies were observed using a stereomicroscope. Then, their lengths were determined and used as phenotypic values. Phenotypic heterosis was evaluated using MP heterosis, which was calculated by the following equation: (F_1_ − MP)/MP × 100%, where F_1_ represents the phenotypic value of the F_1_ hybrid and MP represents the average of the two inbred parents. Moreover, at the maturation stage, mature ears of 10 individuals per line were also harvested and their ear lengths measured.

### Sample preparation, RNA isolation and RNA sequencing

At the 13-leaf stage, young ears were isolated and prepared for RNA-seq. A total of 20 individuals per line from each replicate were mixed for the RNA isolation. Total RNA was extracted using TRIzol reagent. RNA quantity and purity were determined using a Nanodrop 2000 and capillary electrophoresis. All samples with an RNA integrity number greater than 7 were considered of good quality. A total amount of 1 μg RNA per sample was used as input material for the RNA sample preparations. Sequencing libraries were generated using NEBNext® Ultra**™** RNA Library Prep Kit for Illumina® (NEB, USA). In total, 20 RNA samples (10 varieties × 2 replicates) were supplied for deep sequencing using Illumina NovaSeq (150-bp paired-end) at BerryGenomics (Beijing, China), and the raw data included approximately 13–21 million reads per sample.

### Analysis of RNA-Seq data: mapping and quantifying

When the sequencing was completed, Cutadapt 1.10 and in-house Perl scripts were used for quality control [69]. Raw reads were filtered to remove adapters and low-quality bases, as well as reads less than 50 bp in length. Then, HISAT 2.0 [70] was used to map clean reads to the B73 maize reference genome (Version 4), and StringTie 1.3 [71] was used to assemble mapped reads. Finally, using StringTie 1.3 together with Ballgown [72], fragments per kilobase of exon per million (FPKM) mapped sequence reads values were calculated for each sample to estimate the level of gene expression. In addition, the correlation coefficient of the two biological replicates was calculated to evaluate the repeatability of the experiment. The averages of two replicate samples were regarded as the gene expression levels in each line.

### DEG analysis

The criteria (statistical significance) of *p*-value < 0.05 and abs (log_2_ (fold-change) > 1 were used to identify DEGs between two lines with the DESeq package (http://www.bioconductor.org/packages/). Between each two lines (inbred parents and F_1_ hybrids), differential expression analysis was performed. Moreover, the non-additive expression pattern was analyzed in each parent-hybrid triplet. For each triplet, the average expression levels of two parents were calculated as the MP value. Based on the above criteria, non-additively expressed genes were defined as having differential expression levels between those of the F_1_ hybrid and the MP value.

GO enrichment analysis was conducted to determine the essential functions of the DEGs (https://www.omicshare.com/tools). The top 10 GO terms were investigated to determine the major candidate genes. The threshold *p*-value < 0.05 were used for the analysis.

### ASE identification

A specific filter was required for mapping reads. Using a customized Perl script, desired reads that were perfectly mapped to one parental sequence and had single nucleotide polymorphisms mapped to the other were retained. Then, the refiltered reads were assembled according to the previously reported criteria [25]. In each triplet, the refiltered reads from F_1_ hybrid were divided into two sets: set 1, reads aligned against one parent, and set 2, reads aligned against the other parent, to distinguish parent-specific reads in the single nucleotide polymorphism calling step. The normalization of these read numbers was performed using the function estimateSize Factors from the DESeq package [73]. For each gene, ASE was called if the reads of each set deviated significantly from 1:1 by simple random sampling, which was validated by 1000 permutations at a false discovery rate < 0.05.

### Quantitative real-time PCR analysis

The same samples were re-prepared for a quantitative real-time PCR **(**qRT-PCR) analysis in an attempt to validate the expression patterns of key genes. The qRT-PCR was performed in a Bio-Rad CFX96 Real-Time PCR System with SYBR Green PCR Master Mix (Takara Bio). Three technical replicates were included in each plate for qRT-PCR. The *Zm00001d013873* (*ACTIN-2*) gene was used as an internal standard to normalize gene expression, and the relative gene expression levels were measured using the 2^–ΔΔCt^ method [74]. Primers were designed online (https://www.ncbi.nlm.nih.gov/) for the candidate genes, and the primer information was provided in Table S8.

## Supplementary Information

**Additional file 1: Fig. S1**. Schematic drawing of a joint netted pattern including four maize inbred parents and six F_1_ hybrids. Thereinto, two specific maize lines T121 and T126 displayed long ear and short ear, respectively

**Additional file 2: Fig. S2**. Correlation analysis for each RNA-seq replicate

**Additional file 3: Fig. S3**. Parental variation and greater than better-parental variation in gene expression for triplet T121–T126–T121 × T126. **a** Venn diagram comparison between the DEGs in T121 × T126 vs T121 and the key genes responsible for the ear length variation in T121 vs T126. **b** The expression profile of random 10 of those shared genes in a

**Additional file 4: Fig. S4**. Venn diagram comparison between non-additively expressed genes in the F_1_ hybrids and DEGs between the two parents in the six triplets. **a-f** represent triplet T121–PH4CV, T121–PH6WC, T121–T126, T126–PH4CV, T126–PH6WC and PH4CV–PH6WC, respectively. Non- represents non-additive expression

**Additional file 5: Table S1**. The mapped genes and their expression levels

**Additional file 6: Table S2**. The differential expression analysis between different varieties

**Additional file 7: Table S3**. The non-additive expression analysis for the six parent-hybrid triplets

**Additional file 8: Table S4**. The ASE analysis for the six parent-hybrid triplets

**Additional file 9: Table S5**. The GO enrichment analysis of the major genes responsible for the ear length variation between T121 and T126 lines

**Additional file 10: Table S6**. The GO enrichment analysis of the non-additive expression genes for T121 line

**Additional file 11: Table S7**. The GO enrichment analysis of the non-additive expression genes for T126 line

**Additional file 12: Table S8**. The sequence of primers used for qRT-PCR

## Data Availability

The datasets generated and analysed during the current study are available in the NCBI Sequence Read Archive (SRA) database under Bioproject PRJNA682653 (https://www.ncbi.nlm.nih.gov/bioproject/PRJNA682653).

## Abbreviations

DEG: Differentially expressed genes
ASE: Allele-specific expression
RNA-seq: RNA-sequencing
GO: Gene ontology
qRT-PCR: Quantitative real-time pcr
QTL: Quantitative trait loci
TFL1: Terminal flower1
FT: Flowering locus t
SP: Self pruning
MP: Mid-parent
CSN: COP9 signalosome
FPKM: Fragments per kilobase of exon per million
