# Supplementary figures and images for: Global transcriptional profiling between inbred parents and hybrids provides comprehensive insights into ear-length heterosis of maize (Zea mays)
Source: BMC Plant Biol. 2021 Feb 26;21:118. doi: 10.1186/s12870-021-02890-1 (PMC7908659; doi:10.1186/s12870-021-02890-1)

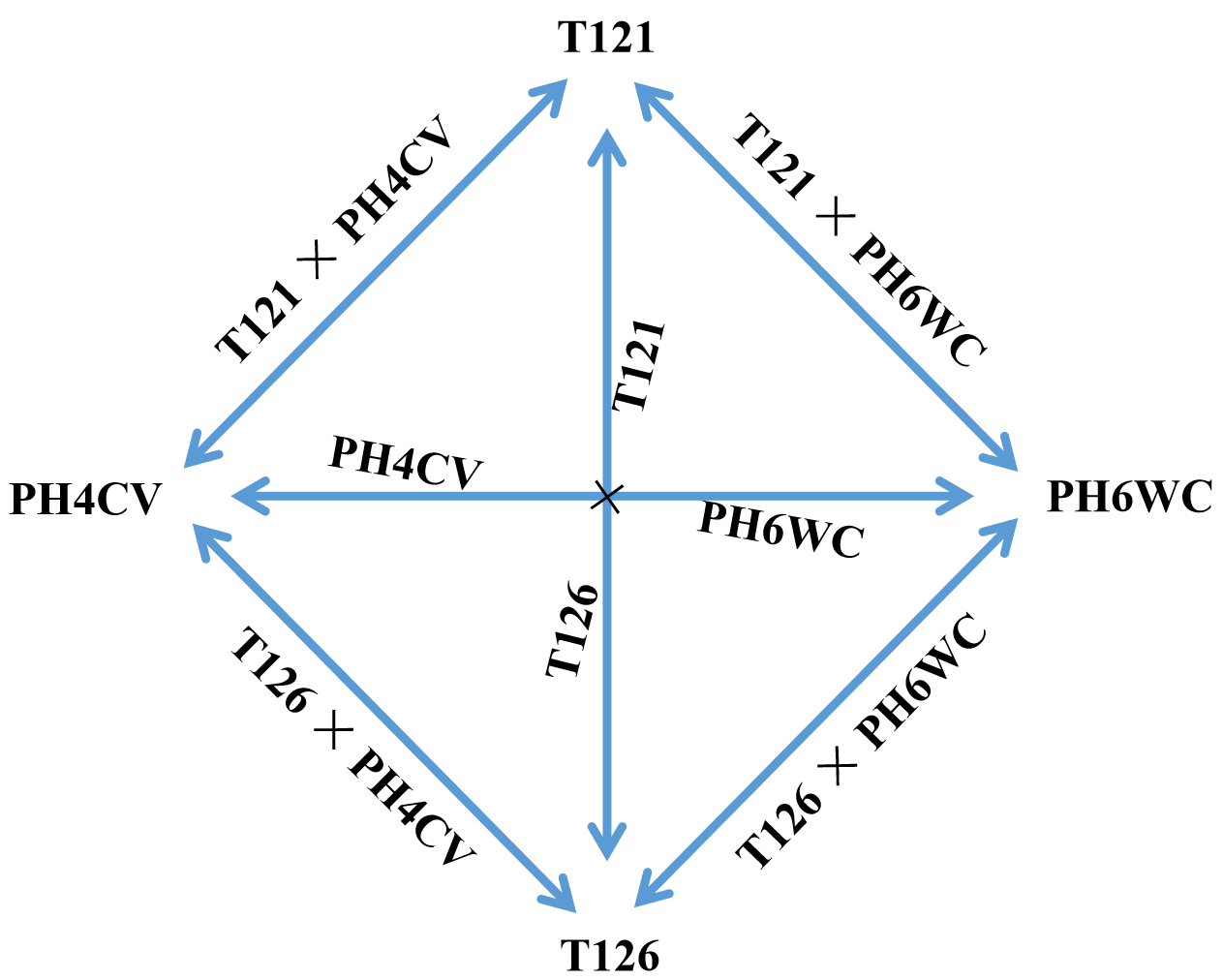

Supplement: Supplementary file 1 — Additional file 1: Fig. S1. Schematic drawing of a joint netted pattern including four maize inbred parents and six F1 hybrids. Thereinto, two specific maize lines T121 and T126 displayed long ear and short ear, respectively [file 12870_2021_2890_MOESM1_ESM.png]

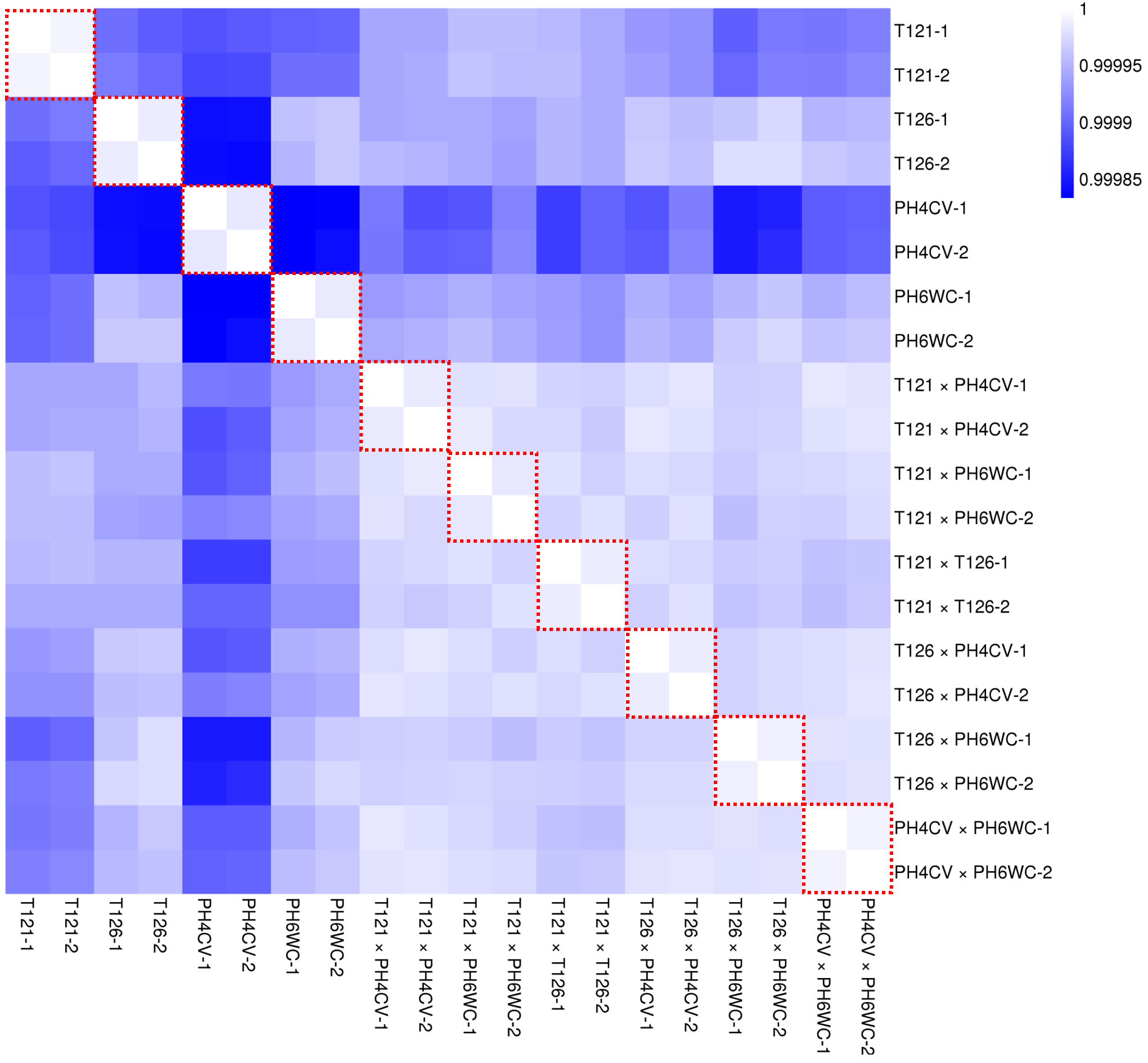

Supplement: Supplementary file 2 — Additional file 2: Fig. S2. Correlation analysis for each RNA-seq replicate [file 12870_2021_2890_MOESM2_ESM.png]

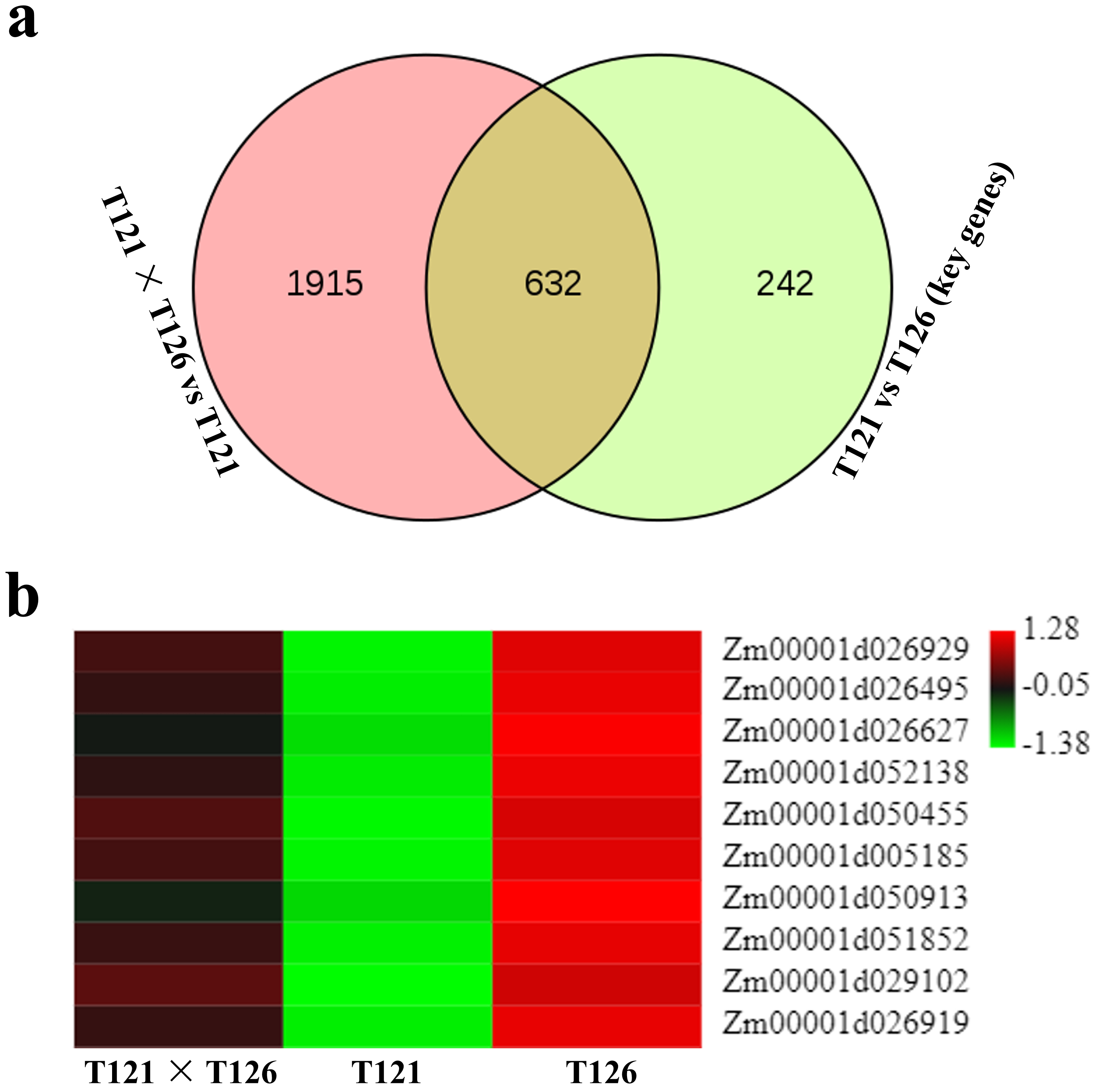

Supplement: Supplementary file 3 — Additional file 3: Fig. S3. Parental variation and greater than better-parental variation in gene expression for triplet T121–T126–T121 × T126. a Venn diagram comparison between the DEGs in T121 × T126 vs T121 and the key genes responsible for the ear length variation in T121 vs T126. b The expression profile of random 10 of those shared genes in a [file 12870_2021_2890_MOESM3_ESM.png]

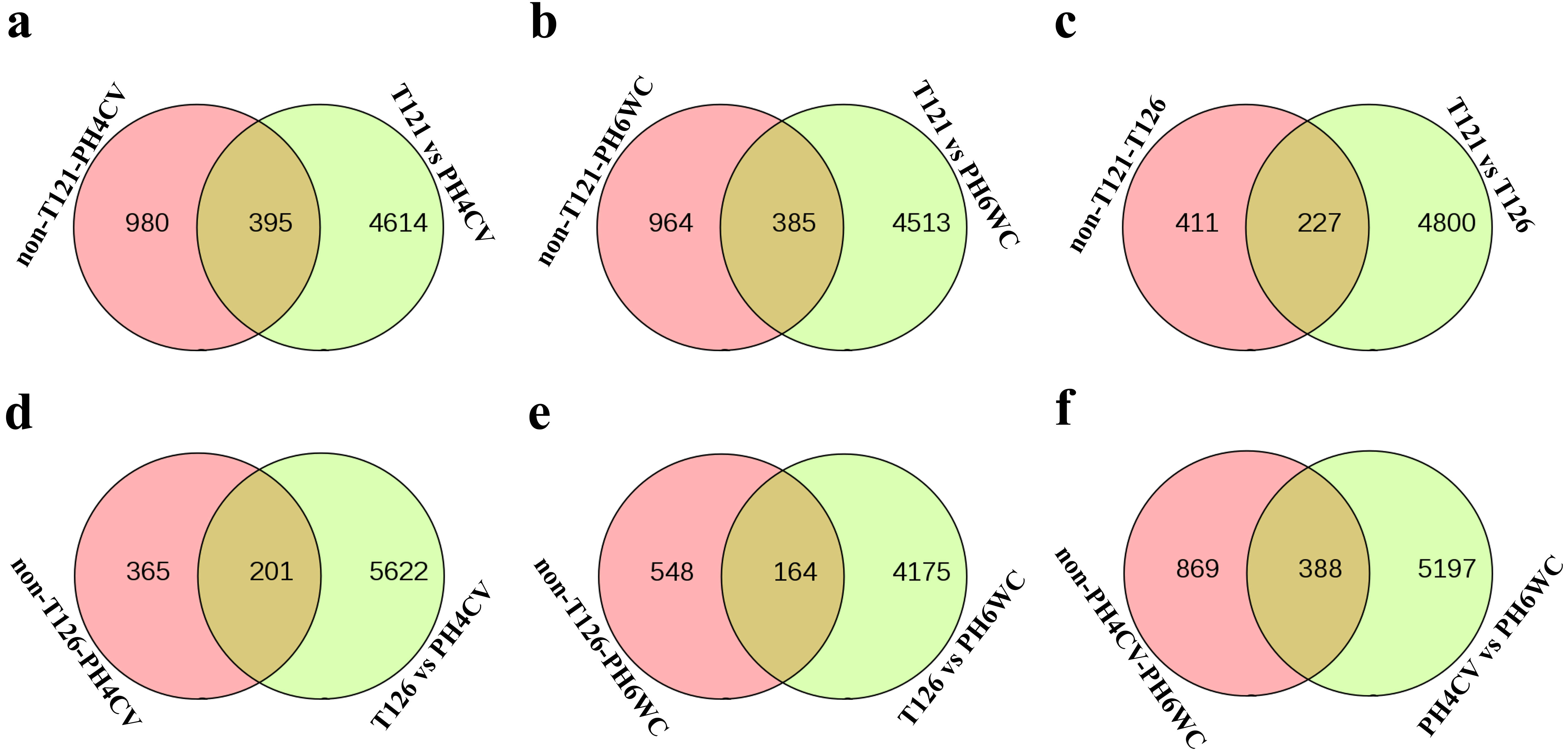

Supplement: Supplementary file 4 — Additional file 4: Fig. S4. Venn diagram comparison between non-additively expressed genes in the F1 hybrids and DEGs between the two parents in the six triplets. a-f represent triplet T121–PH4CV, T121–PH6WC, T121–T126, T126–PH4CV, T126–PH6WC and PH4CV–PH6WC, respectively. Non- represents non-additive expression [file 12870_2021_2890_MOESM4_ESM.png]
