# Supplementary material for: Global transcriptional profiling between inbred parents and hybrids provides comprehensive insights into ear-length heterosis of maize (Zea mays)
Source: BMC Plant Biol. 2021 Feb 26;21:118. doi: 10.1186/s12870-021-02890-1 (PMC7908659; doi:10.1186/s12870-021-02890-1)
Supplement: Supplementary file 12 — Additional file 12: Table S8. The sequence of primers used for qRT-PCR [file 12870_2021_2890_MOESM12_ESM.doc]

Table S8 The sequence of primers used for qRT-PCR

| Genes | Primer-L | Primer-R |
| --- | --- | --- |
| Zm00001d027359 | GTTTTGCCCCCAAACCTAG | TTGATCTCGTCGTAAGCCATC |
| Zm00001d048502 | GAGCTCAACGGATACAGAACTA | GCATAGAAGAAATCGCCAATGT |
| Zm00001d052138 | CAAGAAGCAAGCGTGATCAATA | GACTGCAACATAGAAGCTTGAC |
| Zm00001d049958 | GGTTTCGCCTGAAATCAGATAC | GGACTCGTCATATACACGTCTT |
| Zm00001d050649 | CATCCACAGGTTCATCTTTGTG | GATGGTCTTCTTTCGTACGTTG |
| *ACTIN-2* | TCTTGTAAGATTAGCGGTTTGC | CAGAAGACATGAGTAGCACCTT |
